# Disruption of CCR5 signaling to treat COVID-19-associated cytokine storm: Case series of four critically ill patients treated with leronlimab

**DOI:** 10.1016/j.jtauto.2021.100083

**Published:** 2021-01-06

**Authors:** Nicholas Agresti, Jacob P. Lalezari, Phillip P. Amodeo, Kabir Mody, Steven F. Mosher, Harish Seethamraju, Scott A. Kelly, Nader Z. Pourhassan, C. David Sudduth, Christopher Bovinet, Ahmed E. ElSharkawi, Bruce K. Patterson, Reejis Stephen, Jonah B. Sacha, Helen L. Wu, Seth A. Gross, Kush Dhody

**Affiliations:** a Southeast Georgia Health System, 2415 Parkwood Drive, Brunswick, GA, 31520, USA; b CytoDyn, 1111 Main Street, Suite 660 Vancouver, WA, 98660, USA; c Mayo Clinic, 4500 San Pablo Road, Jacksonville, FL, 3222, USA; d Montefiore Medical Center, Albert Einstein University, 1695A Eastchester Rd, Bronx, NY, 10467, USA; e Spine Center of Southeast Georgia, 1111 Glynco Pkwy Ste 300, Brunswick, GA, 31525, USA; f IncellDx, 1541 Industrial Rd, San Carlos, CA, 94070, USA; g Vaccine and Gene Therapy Institute and Oregon National Primate Research Center, Oregon Health and Science University, 505 N.W. 185th Avenue, Beaverton, OR, 97006, USA; h NYU Langone Gastroenterology Associates, 240 East 38th Street, 23rd Floor New York, NY, 10016, USA; i Amarex Clinical Research, 20201 Century Blvd, Germantown, MD, 20874, USA

**Keywords:** Coronavirus disease 2019 (COVID-19), SARS-CoV-2, Acute respiratory distress syndrome (ARDS), Leronlimab (PRO 140), ACE2, angiotensin-converting enzyme 2, ALT, alanine aminotransferase, ARDS, acute respiratory distress syndrome, AST, aspartate aminotransferase, BID, bis in die (twice a day), CCL2, chemokine C–C motif ligand 2, CCL3, chemokine C–C motif ligand 3, CCL4, chemokine C–C motif ligand 4, CCL5, chemokine C–C motif ligand 5, CCR1, C–C chemokine receptor type 1, CCR5, C–C chemokine receptor type 5, CDC, Centers for Disease Control, CK, creatine kinase, COPD, chronic obstructive pulmonary disease, COVID-19, coronavirus disease 2019, CRP, C-reactive protein, CXCL2, chemokine C-X-C motif ligand 2, CXCL10, chemokine C-X-C motif ligand 10, DPP4, dipeptidyl peptidase-4, DVT, deep vein thrombosis, EDTA, ethylenediaminetetraacetic acid, eIND, emergency investigational new drug application, FDA, Food and Drug Administration, Fi0_2,_ fraction of inspired oxygen, IgG4, immunoglobulin G4, HCQ, Hydroxychloroquine, HLH, hemophagocytic lymphohistiocytosis, HTN, hypertension, ICU, intensive care unit, IL-1β, interleukin 1 beta, IFN-ƴ, interferon gamma, IL-6, interleukin 6, IP-10, interferon gamma-inducible protein (IP) 10 or CXCL10, LOA, letter of authorization, MCP, monocyte chemoattractant protein, M-CSF, macrophage colony stimulating factor, MDC (CCL22), macrophage colony-stimulating factor encoded by the CCL22 gene, MERS-CoV, Middle East respiratory syndrome coronavirus, MIG, monokine induced by IFN-γ (interferon gamma), MIP-1α, macrophage Inflammatory Proteins 1-alpha, MIP-1β, macrophage Inflammatory Proteins 1-beta, N/A, not applicable, NEWS2, National Early Warning Score, NK, natural killer, OSA, obstructive sleep apnea, PDGF-AA, platelet-derived growth factor AA, PDGF-AA/BB, platelet-derived growth factor AA/BB, PEEP, positive end-expiratory pressure, PNA, pulmonary nodular amyloidosis, po, per os (taken by mouth), RANTES, regulated on activation, normal T expressed and secreted (also known as CCL5), RO, receptor occupancy, RT–PCR, reverse transcriptase polymerase chain reaction, SARS-CoV, severe acute respiratory syndrome coronavirus, SARS-CoV-2, severe acute respiratory syndrome coronavirus 2, TGF- α, transforming growth factor alpha, TNF-α, tumor necrosis factor alpha, TNF-β, tumor necrosis factor beta, Tregs, regulatory T cells, T-reg RO, regulatory T cells – receptor occupancy, VEGF-A, vascular endothelial growth factor A, WBC, white blood cell, WHO, World Health Organization

## Abstract

Coronavirus disease 2019 (COVID-19) is associated with considerable morbidity and mortality. The number of confirmed cases of infection with SARS-CoV-2, the virus causing COVID-19 continues to escalate with over 70 million confirmed cases and over 1.6 million confirmed deaths. Severe-to-critical COVID-19 is associated with a dysregulated host immune response to the virus, which is thought to lead to pathogenic immune dysregulation and end-organ damage. Presently few effective treatment options are available to treat COVID-19. Leronlimab is a humanized IgG4, kappa monoclonal antibody that blocks C–C chemokine receptor type 5 (CCR5). It has been shown that in patients with severe COVID-19 treatment with leronlimab reduces elevated plasma IL-6 and chemokine ligand 5 (CCL5), and normalized CD4/CD8 ratios. We administered leronlimab to 4 critically ill COVID-19 patients in intensive care. All 4 of these patients improved clinically as measured by vasopressor support, and discontinuation of hemodialysis and mechanical ventilation. Following administration of leronlimab there was a statistically significant decrease in IL-6 observed in patient A (p=0.034) from day 0–7 and patient D (p=0.027) from day 0–14. This corresponds to restoration of the immune function as measured by CD4+/CD8+ T cell ratio. Although two of the patients went on to survive the other two subsequently died of surgical complications after an initial recovery from SARS-CoV-2 infection.

## Highlights

• Leronlimab is a monoclonal antibody in clinical trials to treat the cytokine storm.
• Critically ill patients received leronlimab through compassionate use and had remarkable recoveries measured objectively.
• The CCR5 receptor is important in recruiting inflammatory cells mainly T cells and macrophages.
• Leronlimab disrupted this signal and may have been responsible for restoration of the immune system, improved survival and decrease in IL-6.

## 1 Introduction

In December 2019, the World Health Organization (WHO) was informed of cases of pneumonia of unknown etiology detected in Wuhan City, Hubei Province, China [1]. On January 12, 2020 the WHO was notified that the outbreak was associated with exposure to a seafood market in Wuhan City [1]. The etiology of these pneumonia cases was subsequently identified as a novel coronavirus, designated severe acute respiratory syndrome coronavirus 2 (SARS-CoV-2). A worldwide coronavirus disease 2019 (COVID-19) pandemic has ensued, with over 76 million confirmed cases reported and over 1.7 million lives lost by late-December 2020 [2]. Among the first hospitalized patients in Wuhan, who had a median age 49 years old, the major causes of mortality were acute respiratory distress syndrome (ARDS), acute cardiac and renal injury, infection and shock [3]. This was an initial warning to the medical community that the use of mechanical ventilation, renal replacement therapy, and intensive care would be necessary for a subset of patients infected with SARS-CoV-2.

The COVID-19 pandemic is one of the world’s worst infectious disease cause of morbidity and mortality since the Spanish Flu of 1918. The Centers for Disease Control (CDC) reported the first case of SARS-CoV-2 infection in the United States in January 2020, and the Secretary of Health and Human Services declared a public health emergency on January 31, 2020 in response to COVID-19 [4]. A proclamation declaring a national emergency concerning the COVID-19 outbreak was released on March 13, 2020 as healthcare providers in the United States braced for the pandemic [5]. Due in part to the unknown implications of COVID-19, the Coronavirus Aid, Relief, and Economic Security (CARES) Act was passed by Congress to provide emergency economic assistance and limit health provider liability during this public health emergency [6]. COVID-19 treatment protocols have continued to change based on CDC guidelines and have included prone positioning and first the use, then removal of hydroxychloroquine as a treatment option due to increases in death rates, and more recently the potential role of other treatments including the use of remdesivir and corticosteroids in the management of COVID-19 [[7], [8], [9], [10], [11]].

The current pandemic should not come as a surprise to us, *Betacoronavirus* family members that have caused prior epidemics include severe acute respiratory syndrome coronavirus (SARS-CoV) in 2002–2003 and Middle East respiratory syndrome coronavirus (MERS-CoV) in 2012. These viruses have many common characteristics such as specific host cell receptors, genomic sequence, reservoir, risk factors, and etiology of severe disease (Table 1). SARS-CoV and SARS-CoV-2 share the common angiotensin-converting enzyme 2 (ACE2) host cell receptor, while MERS-CoV binds to the dipeptidyl peptidase-4 (DPP4) receptor. The genomic homology between SARS-CoV-2 and SARS-CoV and MERS-CoV is approximately 79% and 50%, respectively, and was the first step to identify pathophysiology and potential treatment targets [12]. Risk factors for poor outcomes associated with the three different *Betacoronaviruses* include advanced age, cardiovascular disease, pulmonary disease and cancer [13,14]. Acute respiratory distress syndrome (ARDS) due to SARS-CoV-2 infection results from immune hyperactivation, also referred to as cytokine storm [15]. The inflammatory cytokines and chemokine upregulation observed in lungs at autopsy is due to the activation of macrophages, dendritic cells and T cells that lead to multisystem organ failure. The cytokines interleukin 6 (IL-6), interleukin 1 beta (IL-1β), tumor necrosis factor alpha (TNF-α) and chemokine C–C motif ligand 2 (CCL2), 3 (CCL3), and 5 (CCL5) are upregulated and represent potential targets for treatment of patients with COVID-19 [16].

**Table 1:** MERS-CoV, SARS-CoV, and SARS-CoV-2 characteristics.

	MERS-CoV	SARS-CoV	SARS-CoV-2
Host cell entry receptor	DPP4	ACE2	ACE2
Shared genome with SARS-CoV-2 [12]	50%	79%	N/A
Reservoir [52]	Bats	Bats	Bats
Risk factors for poor outcomes	Advanced age, male sex, DM, HTN, cancer, lung disease [12]	Advanced age, male sex [55]	DM, HTN, advanced age, cardiovascular disease, cancer [13,14]
	Cytokine storm,		Cytokine storm,
	C5a-induced CD8^+^ T cell reduction [53,54]	Cytokine storm, reduction in CD8^+^ T cells	reduced CD4+/CD8+ T cell ratio [56]
Severity of disease etiology			

ACE2, angiotensin-converting enzyme 2; C5a, complement component 5a; DM, diabetes mellitus; DPP4, dipeptidyl peptidase-4; HTN, hypertension; N/A, not applicable.

Due to the rapid surge in deaths in patients with cytokine storm due to SARS-CoV-2 infection, traditional drug discovery and development approaches have been replaced with a drive to rapidly repurpose potential therapies with established safety profiles. These have largely focused on therapeutic agents with potential antiviral activity against SARS-CoV-2 such as lopinavir–ritonavir [17], remdesivir [[18], [19], [20], [21]], and hydroxychloroquine [10,22,23] and a variety of immunological therapeutics, including dexamethasone [24], targeting the immune dysregulation caused by SARS-CoV-2. Among the immunological therapies monoclonal antibodies are potentially, the most promising. These repurposed monoclonal antibody drugs were initially developed to treat conditions such as inflammatory bowel disease, rheumatoid arthritis, other autoinflammatory diseases, and primary hemophagocytic lymphohistiocytosis (HLH) [[25], [26], [27], [28], [29], [30]]. To date, these trials have been largely unsuccessful with most showing no or limited benefits, however, perhaps the most troubling aspect is that they have shown only at most modest benefit in the most severely and critically ill patients.

Leronlimab (PRO 140) is a humanized IgG4, C–C chemokine receptor type 5 (CCR5), monoclonal antibody receptor antagonist originally studied in the treatment of HIV for which it has been shown to be effective and well tolerated [[31], [32], [33], [34], [35]] and currently being investigated for the treatment of COVID-19. Leronlimab is the only monoclonal antibody thus far to demonstrate improvements in National Early Warning Score (NEWS2) in patients with mild to moderate COVID-19 [36]. Leronlimab is administered to COVID-19 patients via subcutaneous injection 700 ​mg once a week for two weeks. The CCR5 receptor is located on macrophages, T regulatory cells and dendritic cells. The binding of leronlimab to the CCR5 receptor competitively inhibits CCL5 [37]. It therefore decreases downstream release of proinflammatory cytokines, while also inhibiting migration of regulatory T cells to the site of infection [34,38]. Leronlimab was also seen as a potential treatment option for COVID-19 due to its good safety profile in patients with HIV, mechanism of action, and basic science physiology [[31], [32], [33], [34], [35]]. Leronlimab was studied in 2001 by Trkola *et al* and was found to be a CCR5 antagonist that binds to, but does not transduce signals on T cells and macrophages [39]. Multiple clinical trials in at-risk HIV patient populations have revealed no serious adverse events or deaths related to the use of leronlimab [[31], [32], [33], [34], [35]].

## 2 Methods

Four patients were admitted to the intensive care unit with confirmed nasopharyngeal swabs positive for SARS-CoV-2 infection by reverse transcriptase polymerase chain reaction (RT–PCR). Amarex, the clinical research organization contracted by CytoDyn provided the health system with a letter of authorization (LOA), pharmacy manual, informed consent, and treatment protocol. The LOA was submitted to the FDA after receiving informed consent for compassionate use of leronlimab. Due to current COVID-19 pandemic contact restrictions, telephone consent with intensive care unit nurse confirmation was documented and three emergency investigational new drug applications (eIND) were submitted and approved by the FDA on April 15, 2020. A fourth eIND was submitted on May 28, and leronlimab was administered on June 2, 2020.

Per the leronlimab treatment protocol schedule of assessments, a physical exam, vital signs, positive end-expiratory pressure (PEEP) along with an electrocardiogram and required laboratory data were collected. Two samples in ethylenediaminetetraacetic acid (EDTA) tubes were sent to IncellDx to determine cytokine and chemokine concentrations, CCR5 receptor occupancy on regulatory T cells (Tregs), monocyte and T cell counts as well as CD4%, CD8% and ratio of CD4+/CD8+ T cells before injection of leronlimab and at Day 3, 7, and 14 post-injection. Pharmacy staff prepared the dose and the intensive care unit nurses administered the drug subcutaneously while wearing personal protective equipment per CDC guidelines. Three patients received their first dose of leronlimab on April 17, 2020 and another on June 2, 2020. Throughout the hospital course, patients’ information was collected via physician physical examination, leronlimab protocol blood draws, and chart review. Assessment of plasma cytokines and chemokine levels were analyzed using a flow cytometric assay (LEGENDplex™, BioLegend Inc, CA, USA). Peripheral blood mononuclear cells were analyzed using Lymphoprep™ density gradient (STEMCELL Technologies, Vancouver, Canada). CCR5 receptor occupancy by leronlimab was detected by flow cytometry through labeling technology using phycoerythrin-labeled leronlimab. The data described here are current through December 2020.

Statistical analysis of the cytokines IL-6 and TNF-α were compared to a control by the non-parametric Kruskal–Wallis test and Dunn’s multiple comparison correction. To determine a shift towards immune system homeostasis after administration of leronlimab, the Kruskal–Wallis test with Dunn’s multiple comparison correction was used. We report p-values generated through the GraphPad Prism computer program.

## 3 Case series

The novel coronavirus SARS-CoV-2 was identified in four critically ill patients who developed ARDS. Treatment target was the cytokine storm created by SARS-CoV-2 infection. After receiving leronlimab, all four patients initially survived. Two patients went on to recover and were discharged from hospital, while the other two patients subsequently died of surgical complications after making an initial recovery from SARS-CoV-2 infection. All four patients clinically improved as measured by vasopressor support, and discontinuation of hemodialysis and mechanical ventilation. None of the patients developed a thromboembolic event after leronlimab injection. Demographics including age, date of hospital admission, date of intubation, days intubated before administration of leronlimab, significant past medical history, duration of symptoms before hospital admission, and the use of investigational drugs administered before leronlimab are listed in Table 2. The average age of these four patients was 60 years. Three females were hospitalized in early April and one male was hospitalized at the end of May. The mean time to intubation from date of hospitalization was 2 days. The number of days the patients were intubated prior to receiving leronlimab ranged from 4 to 15 days, with a mean of 7 days. The most common comorbidity was being a former or current smoker. Two of the four patients had a history of chronic obstructive pulmonary disease (COPD). Patients A and D had a concurrent diagnosis of right upper extremity deep vein thrombosis (DVT) before administration of leronlimab; none of the patients developed a thromboembolic event after leronlimab injection. Patients B, C, and D developed acute kidney injury requiring hemodialysis, while patient A did not. The mean duration of symptoms before hospitalization was 7 days (range 2–10 days).

**Table 2:** Patient demographics.

Parameter	Patient A	Patient B	Patient C	Patient D
Age of patient, years	58	50	58	75
Date of hospital admission	4-12-20	4-7-20	4-1-20	5-28-20
Date of intubation	4-13-20	4-12-20	4-2-20	5-29-20
Date of confirmed COVID-19	4-12-20	4-11-20	4-7-20	5-28-20
Days intubated before leronlimab treatment				
Date(s) of leronlimab treatment	4	5	15	4
Comorbidities	Day 0: 4-17-20	Day 0: 4-17-20	Day 0: 4-17-20	Day 0: 6-2-20
	Day 7: 4-24-20	Dose 2 not given	Day 18: 5-5-20	Day 10: 6-10-20
	Migraines, ex-smoker (20 pack years, quit 2 years ago)	COPD, OSA	Asbestosis, COPD, ex-smoker (50 pack years), HTN, DM, hypercholesterolemia	Bradycardia, beta thalassemia
	DVT		Acute kidney injury, sacral decubitus ulcer	
Concurrent diagnoses during admission		Necrotizing fasciitis of right forearm, acute kidney injury, DVT		DVT, PNA
		Current smoker (2 packs per day)	Ex-smoker	None
		7		
Smoking history	Ex-smoker	HCQ 200 ​mg po BID x 5 days	10	2
		10		Tocilizumab, remdesivir
Onset of symptoms before hospitalization, days	10	4	Zinc, HCQ, azithromycin	Deceased
Other investigational drugs administered	HCQ 200 ​mg po BID x 5 days	17	Deceased	N/A
Total days in ICU	21	Discharged 5-26-20	N/A	
Days in ICU after leronlimab treatment	18	50		N/A
Removed from oxygen after receiving leronlimab, days	19		N/A	
Discharged status	Discharged 5-19-20		N/A	N/A
Total days in hospital	38		N/A	N/A

BID, twice daily; COPD, chronic obstructive pulmonary disease; DM, diabetes mellitus; DVT, deep vein thrombosis; HCQ, hydroxychloroquine; HTN, hypertension; ICU, intensive care unit; N/A, not applicable; OSA, obstructive sleep apnea; po, taken by mouth; PNA, pneumonia.

Investigational drugs the patients received during their hospital course included hydroxychloroquine, zinc, tocilizumab, remdesivir, and azithromycin. Total days in the intensive care unit (ICU) and total days in the ICU after treatment with leronlimab could only be calculated for patients A and B, which were 21 and 10 days, and 18 and 4 days, respectively. Patient C developed an unstageable ulcer and died due to complications after a diverting ostomy in an effort to prevent wound infection. Before his death patient C was on daily hemodialysis once per week with improved creatinine clearance. Before the deaths of patients C and D, neither required vasopressors; patient C had a tracheostomy and was on the ventilator 12 ​h per day, while patient D had been extubated for 24 ​h and was re-intubated due to tachypnea. Patient D clinically improved and at the time of death was on weaning parameters for extubation. Patient D died due to iatrogenic perforation of the colon during the placement of a gastrostomy tube. Patient A and patient B were respectively removed from oxygen on Day 19 and Day 17 and discharged from the hospital 38 and 50 days after admission.

**Fig. 1:**
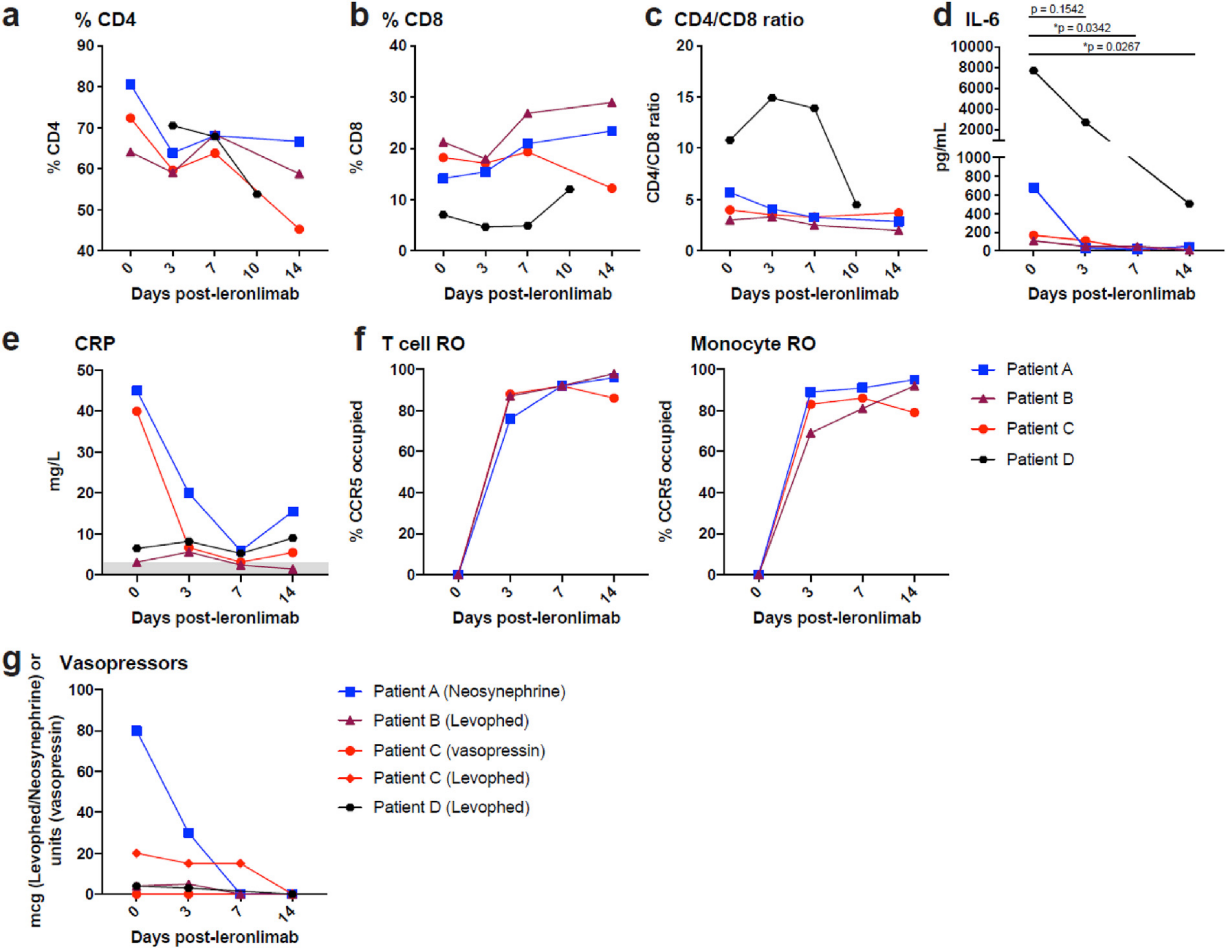
Immune restoration as measured by CD4% (a), CD8% (b), and CD4+/CD8+ T cell ratio (c); IL-6 levels (d); CRP levels (e); T cell and monocyte receptor occupancy (f); and vasopressor use (g) at Days 3, 7, and 14.

For each of the four patients, cytokine profiles were examined before administration of leronlimab (Day 0), then on Days 3, 7, and 14. IL-6 and TNF-α levels, T cell and macrophage receptor occupancy of leronlimab, CRP levels, baseline ventilatory settings, and vasopressor requirements were recorded and are shown in Table 3 and Fig. 1. Complete blood counts, metabolic and coagulation profiles on the first day of leronlimab treatment are presented in Table 4. Additional cytokine profiles for patients are provided in Supplemental Figs. A and B. For patients A and D, there was a statistically significant decrease in IL-6 at day 7 (p=0.034) and 14 (p=0.027), respectively, when compared to baseline, after dosing with leronlimab (Fig. 1d). Furthermore, we observed a decrease in TNFα in both patient A and B after leronlimab injection. Restoration of immune cell function as measured by the CD4+/CD8+ T ratio was observed between Days 7 and 14 (Fig. 1a, b, and c). Patient A had a CRP of 45 ​mg/L at Day 0, which decreased to 5.9 ​mg/L by Day 7. In patient C, CRP decreased from 40 ​mg/L at Day 0–3.1 ​mg/L on Day 7 (Fig. 1e). CRP in patient B was normal, while it was twice the upper limit of normal in patient D. The T cell and macrophage occupancy of the CCR5 receptor was zero for each patient before leronlimab injection. The median occupancy on Days 3, 7, and 14 for patients A, B and C was approximately ≥80% (Fig. 1f). Additional cytokine profiles included macrophage colony stimulating factor (M-CSF), which was elevated in patient B, but normal in the others as reported on Days 0, 3, and 7 (Supplemental Fig. B).

**Table 3:** Chemokine and cytokine levels, leronlimab receptor occupancy, CRP levels, ventilatory settings, and vasopressor requirements.

	Day 0	Day 3	Day 7	Day 14
Patient A				
IL-6, pg/mL (normal range 5–15 ​pg/mL)	679.8	32.3	19.3	46.7
TNF-α, pg/mL (normal range <3 ​pg/mL)	79.5	28.7	16.1	218.5
RANTES, pg/mL (normal range <500 ​pg/mL)	847	1730	2131	410
CD4%	80.6	63.9	68.1	66.7
CD8%	14.1	15.4	20.9	23.4
CD4+/CD8+ T cell ratio (normal value 2.0)	5.70	4.10	3.26	2.85
T cell RO, %	0	76	92	96
Monocytes RO, %	0	89	91	95
Tregs RO, %	0	87	94	98
CRP, mg/L (normal range 3–5 ​mg/L)	45	20	5.9	15.5
PEEP, cmH_2_0	14	12	8	5
Fi0_2_, %	90	60	50^a^	40
Vasopressor				
Neosynephrine, mcg	80	30	N/A	N/A
Patient B^b^				
IL-6, pg/mL (normal range 5–15 ​pg/mL)	108.67	49.36	48.73	11.7
TNF-α, pg/mL (normal range <3 ​pg/mL)	734.1	173.55	132.4	11
RANTES, pg/mL (normal range <500 ​pg/mL)	2986	2328	193	177
CD4%	64.1	59.1	68.4	58.8
CD8%	21.2	17.9	26.8	28.9
CD4+/CD8+ T cell ratio (normal value 2.0)	3	3.3	2.5	2
T cell RO, %	0	87	92	98
Monocytes RO, %	0	69	81	92
Tregs RO, %	0	83	95	99
CRP, mg/L (normal range 3–5 ​mg/L)	3.1	5.6	2.4	1.5
PEEP, cmH_2_0	5^c^	n/a	n/a	n/a
Fi0_2_, %	40	4 ​L O_2_ (NC)	4 ​L O_2_ (NC)	2 ​L O_2_ (NC)
Vasopressor				
Norepinephrine bitartrate, mcg	4	5^d^	N/A	N/A
Patient C^e^				
IL-6, pg/mL (normal range 5–15 ​pg/mL)	170	111	13	49
TNF-α, pg/mL (normal range <3 ​pg/mL)	70	55	37	27
RANTES, pg/mL (normal range <500 ​pg/mL)	475	1507	1563	2122
CD4%	72.4	59.7	63.8	45.3
CD8%	18.2	17.0	19.3	12.2
CD4+/CD8_ T cell ratio (normal value 2.0)	3.99	3.50	3.30	3.70
T cell RO, %	0	88	92	86
Monocytes RO, %	0	83	86	79
Tregs RO, %	0	92	93	84
CRP, mg/L (normal range 3–5 ​mg/L)	40	6.7	3.1	5.5
PEEP, cmH_2_0	14	14	14	10
Fi0_2_, %	90	70	70	60
Vasopressors^f^				
Norepinephrine bitartrate, mcg	20	15	15	N/A
Vasopressin, units	0.04	0.04	N/A	N/A
Patient D				
IL-6, pg/mL (normal range 5–15 ​pg/mL)	7678.0	2689.0	Missing	506.7
TNF-α, pg/mL (normal range <3 ​pg/mL)	168.6	152.7	Missing	91.4
RANTES, pg/mL (normal range <500 ​pg/mL)	2068.0	2586.0	Missing	1227.0
CD4%		70.5	67.9	53.8
CD8%	7.0	4.7	4.9	12.0
CD4+/CD8_ T cell ratio (normal value 2.0)	10.8	14.9	13.9	4.5
T-cell receptor occupancy (%)	0	68	88	90
T-reg receptor occupancy (%)	0	74	82	92
CRP, mg/L (normal range 3–5 ​mg/L)^i^	6.5	8.2	5.3	9.0
PEEP, cmH_2_0	8	8	8	6
Fi0_2_, %	70	60	50^h^	90
Vasopressor				
Norepinephrine bitartrate, mcg	4	3^g^	N/A	N/A

CRP, C-reactive protein; Fi0_2_, fraction of inspired oxygen; IL-6, interleukin 6; N/A, not applicable; N/C, nasal cannula; PEEP, positive end-expiratory pressure; RANTES, regulated upon activation, normal T cell expressed and presumably secreted; RO, receptor occupancy; TNF-α, tumor necrosis factor alpha; Tregs, regulatory T cells.

a Patient self-extubated on Day 9 and was re-intubated on day 11 due to lethargy.

b Patient refused second dose of leronlimab.

c Patient extubated on 4-18-20.

d Norepinephrine bitartrate stopped 4-23-20.

e Patient received second dose of leronlimab on 5-5-20 due to clinical condition and possible withdrawal of care.

f Vasopressin discontinued on day 4 and norepinephrine bitartrate discontinued on day 9.

g Off norepinephrine bitartrate on Day 5.

h Extubated on Day 9 and reintubated on Day 11.

i Patient received tocilizumab on Days 5–29 and a 5-day remdesivir course.

**Table 4:** Complete blood counts, complete metabolic profiles, and coagulation profiles on the first day of treatment with leronlimab (Day 0).

Parameter	Patient A	Patient B	Patient C	Patient D
Hemoglobin, g/dL (normal range 12–15 ​g/dL)	6.5	11	8.5	10
Hematocrit, g/dL (normal range 36–38 ​g/dL)	21	34	30	31
Platelet count, cells X 10^9^/L	286	357	135	296
WBC, cells X 10^9^/L	6.6	25.6	22	8.8
PT, sec (normal range 11–13.5 ​s)	15.9	13.9	17.6	14.5
INR (normal range 0.8–1.1)	1.3	1.1	1.5	1.1
Glucose, mg/dL (normal range 70–100 ​mg/dL)	133	125	276	120
Blood biochemistry				
Na, mEq/L	137	136	134	133
K, mmol/L	4.2	4.1	4.7	3.4
Cl, mEq/L	104	103	100	99
CO_2_, mmol/L	26	28	23	33
BUN, mg/dL	15	17	18	24
Cr, mg/dL	0.5	1.0	1.0	0.50
LFTs				
Total Bilirubin, mg/dL	0.2	1.2	1.5	0.3
ALP, IU/L	101	566	166	79
AST, IU/L	41	119	146	43
ALT, IU/L	10	157	20	17
Lactic acid (normal range 0.5–1 ​mmol/L)	1.5	0.5	2.2	2.0
Ferritin (normal range 12–300 μg/L)	133	133	323	457
Metabolic profile				
pH	7.30	7.28	7.15	7.46
CO_2_, mmol/L	49	52	68	38
O_2_, mmHg	107	93	152	81
HCO_3_, mEq/L	24	24	24	27
A-a gradient, mmHg	473	126	478	370
Urinalysis			N/A (on dialysis)	
pH	6	5.5		6
Protein	1+	2+		Trace
Ketones	0	Trace		–
Blood	0	2+		–
CK (normal range 22–198 U/L)	32	20	353	220

ALP, alkaline phosphatase; ALT, alanine transaminase; AST, aspartate transaminase; BUN, blood urea nitrogen; CK, creatine kinase; Cl, chloride; CO_2_, carbon dioxide; Cr, creatinine; HCO_3_, bicarbonate; INR, International Normalized Ratio; K, potassium; LFT, liver function test; NA, sodium; N/A, not applicable; O_2_, oxygen; PT, prothrombin time; sec, seconds; WBC, white blood cell.

Per the leronlimab treatment protocol, baseline laboratory parameters such as complete blood counts, complete metabolic profile, and coagulation profiles were assessed before administration of leronlimab (Table 4). Three of the four patients had anemia, with baseline mean hematocrit 29 ​g/dL (range 21–34 ​g/dL). The mean white blood cell (WBC) count was 15.75 ​× ​10^3^/μL (range 6.6–25.6 ​× ​10^3^/μL). Thrombocytopenia at baseline was seen in patient C. All patients had some degree of abnormal liver chemistries. The most significant changes were seen in patients B and C who had aspartate aminotransferase (AST) and alanine aminotransferase (ALT) elevations 2–7 X the upper limit of normal and alkaline phosphatase elevations 4.3 X the upper limit of normal or 566 U/L. Lactic acid levels in patients A, C, and D were mildly elevated at 1.5, 2.2 and 2.0 ​mmol/L, respectively (normal range 0.5–1 ​mmol/L). A-a gradients were markedly abnormal in all patients. Urinalysis could not be obtained in patient C as he was anuric. Patients C and D had abnormal serum ferritin measurements and elevated creatine kinase (CK) levels.

Clinically, patient B had the smallest oxygen requirement, was on one vasopressor, and had a concurrent diagnosis of necrotizing fasciitis status post debridement of her wound 5 days prior to receiving leronlimab. She was extubated within 24 ​h of receiving leronlimab. CRP levels decreased in patients A and C; IL-6 and TNF-α alpha levels decreased in all three patients. Patient B had near normal CRP levels since admission, but pathologic cytokines TNF-α and IL-6 were elevated significantly.

## 4 Discussion

Host cell defenses to viral infections are based on chemokine and cytokine signals. Pathologically overproduced chemokines and their respective receptors have been identified in patients with COVID-19. In a landmark study, Chua *et al* described lung epithelium and immune cell interactions and concluded that pharmacological inhibition of either the C–C chemokine receptor type 1 (CCR1) or CCR5 pathways may restore immune homeostasis [40].

Although the exact mechanism of chemokine inhibition by leronlimab, a CCR5 receptor antagonist, is not fully understood, a proposed mechanism based on prior studies of the CCL5/CCR5 axis has been established. The chemokine CCL5 has been well studied in recruitment of activated natural killer (NK) cells and CD8^+^ T cells as well as macrophages in respiratory infections and malignancy [41,42]. Along with altered chemotaxis created by unregulated CCL5, cytokines such as IL-6 and TNF-α continue to cause an inflammatory cascade leading to ARDS and multisystem organ failure [[42], [43], [44], [45]].

Our patients had a restoration of immune system function as measured by their normalized CD4+/CD8+ T cell ratio. In a study published in *Immunity* in 2008, Kohlheimer *et al* reported that mice expressing the CCR5 receptor had more CD8^+^ T cells recruited to the lungs when infected with influenza and parainfluenza compared with mice that were CCR5 deficient [46]. The chemotaxis effect of the CCR5 receptor accelerated the recruitment of CD8^+^ T cells to attack viruses. Other chemokine ligands associated with chemotaxis of the CCR5 receptors in this study were CCL3, chemokine C–C motif ligand 4 (CCL4) and CCL5 [46]. This study also showed that the proinflammatory cytokines such as interferon gamma (IFN-ƴ), TNF-α, IL-6, CCL2, and chemokine C-X-C motif ligand 2 (CXCL2) were all elevated in mice expressing the CCR5 receptor.

The downstream effect of altering the cytokine storm signal may have decreased the release of inflammatory cytokines such as IL-6, TNF-α, and CRP. Levels of IL-6 and TNF-α were inversely related to the patients’ clinical conditions. CRP was a marker for decrease in inflammation and improved clinical condition in two patients at Day 7. Until further clinical studies are performed, one should be cautious to use this as a surrogate marker in severe SARS-CoV-2 infection unless the initial CRP level is elevated. The near normal CRP level in patient D is likely due to the patient receiving tocilizumab, an IL-6 receptor antagonist. In patient B, accounting for the near normal CRP level in the setting of necrotizing fasciitis (status post debridement less than 24 ​h before her first dose of 700 ​mg of leronlimab), acute renal failure and acute hypoxemic respiratory failure cannot account for this value. It is well documented in the literature that CRP measurement as a prognostic indicator in immunocompromised surgical patients is 69% sensitive and 76% specific for the diagnosis of a concurrent infection; it should be combined with clinical decision making when evaluating post-surgical patients [47]. CRP may be used in the future as a surrogate marker for clinical improvement depending on ongoing clinical trial data. If cytokine profiles are not available, CRP may be a surrogate biomarker to predict the need for vasopressor support in critically ill patients when baseline CRP level is elevated.

Our observations of clinical improvement in a group of heterogenous critically ill patients were objectively recorded by measure of vasopressor support and ventilatory changes. All patients had a prolonged hospital course, and all patients not only clinically improved but required less life sustaining resources such as mechanical ventilation and hemodialysis. We believe patient A had the classic cytokine storm as measured by markedly elevated CRP level and statistically significant decrease in IL-6 level after leronlimab administration. Although the patient developed a DVT prior to administration of leronlimab, she did not develop renal failure or any further thromboembolic events prior to discharge. On Day 3, the patient’s vasopressor support, PEEP, and fraction of inspired oxygen (Fi0_2_) decreased until she self-extubated on Day 9. She was reintubated due to lethargy and again extubated on Day 11, and was removed from oxygen by Day 19, spending a total of 38 days in the hospital. Patient B was admitted to the intensive care unit for respiratory distress and was extubated 24 ​h after receiving leronlimab. She developed renal failure requiring hemodialysis due to COVID-19 and, although she did not receive a second dose of leronlimab, she was no longer on renal replacement therapy at the time of discharge nor did she have any further thromboembolic events. Patient C was by far the most critically ill. He was intubated for 15 days prior to dosing with leronlimab. Ventilatory settings were PEEP 14 and Fi0_2_ 90%; the patients was on two vasopressors. Twenty-four hours after administration of leronlimab, the patients’s Fi02 was decreased from 90% to 80%, and norepinephrine bitartrate was decreased from 20 to 18 mcg. Vasopressin was discontinued by day 4, and norepinephrine bitartrate was discontinued on Day 9. Patient D followed the same clinical course as patient A, and although she died of iatrogenic bowel perforation during gastrostomy tube placement, she was on minimal ventilatory settings (PEEP 5, Fi0_2_ 40%) at the time care was withdrawn.

Although patients with COVID-19 may follow the same clinical course, they may have unique immunological presentations. Divij *et al* showed that half of patients with confirmed SARS-CoV-2 infection had a decrease in CCL5 and IL-6 levels and an increase in chemokine C-X-C motif ligand 10 (CXCL10), interleukin-1 receptor agonist (IL-1RA), and CCL2 levels [48]. This corresponds to the cytokine profiles of our patients. The CCL5–CCR5 axis is complex, and although restoration of the immune system by measure of the CD4+/CD8+ T cell ratio is one potential mechanism of action of leronlimab, another may be related to macrophage survival and virus removal according to Marques et al. Virus-infected CCR5-and CCL5-deficient mice produced apoptotic signals in macrophages containing virus [49]. Enhanced survival of macrophages through inhibition of the CCR5–CCL5 axis by leronlimab may regulate removal of virus and cellular debris and aid in decreasing the inflammatory cascade [49]. High concentrations of CCL5 not only cause T cell activation, but at low levels leads to T cell recruitment and is protective against macrophages [50]. Although this model was based on knockout mice and not on CCR5 blockade, it provides insight into another possible mechanism of action and further studies may confirm our hypothesis. Varied levels of RANTES may represent the heterogeneity of this disease and the specific virologic or immunological state of the patient. The lower levels of CCL5 or RANTES in our patients underscores a paradoxical protective mechanism, which may aid in viral clearance by macrophages.

## 5 Conclusion

At the time we used leronlimab for the treatment of COVID-19, reported outcomes were limited to eIND patients. Subcutaneous administration of leronlimab was safe and may have been associated with remarkable recoveries in the four critically ill patients with SARS-CoV-2 infection, two of whom went on to be discharged from hospital while the other two died of surgical complications after their initial recovery from SARS-CoV-2 infection. CCR5 receptor blockade with leronlimab was associated with a statistically significant decrease in IL-6 (p=0.034) and restoration of the immune function as measured by CD4+/CD8+ T cell ratio. The newest literature supports the use of a CCR5 or CCR1 receptor antagonist for patients infected with SARS-CoV-2 [40]. All four of our patients clinically improved, but unfortunately two died of surgical procedures unrelated to the use of leronlimab. None of the patients that have received leronlimab developed DVT, and all of the patients requiring hemodialysis were no longer dialysis dependent at the time of discharge. Leronlimab is currently being studied in multi-center, double-blind, randomized controlled clinical trials in the US in patients with critical SARS-CoV-2 infection [51]. The previous clinical trial to treat patients with mild-to-moderate SARS-CoV-2 infection demonstrated a statistically significant improvement in NEWS2 assessment compared to placebo [36]. In this trial leronlimab was also associated with fewer and less severe adverse events than placebo. Emergency use authorization is currently pending as well as forthcoming Phase 3 clinical trial data.

## Declaration of competing interest

The authors declare that they have no known competing financial interests or personal relationships that could have appeared to influence the work reported in this paper. Dr. Kelly is the chief medical officer of Cytodyn and Dr Pourhassan is the chief executive oficer of Cytodyn and have a financial interest in Cytodyn but did not influence the work reported in this paper. Dr. Dhody is the Chief research officer for Amarex and may have a financial interest in Cytodyn but did not influence the work reported in this paper. Dr Sacha is a consultant for Cytodyn but did not influence the work reported in this paper.
