# Supplementary figures and images for: Disruption of CCR5 signaling to treat COVID-19-associated cytokine storm: Case series of four critically ill patients treated with leronlimab
Source: J Transl Autoimmun. 2021 Jan 6;4:100083. doi: 10.1016/j.jtauto.2021.100083 (PMC7823045; doi:10.1016/j.jtauto.2021.100083)

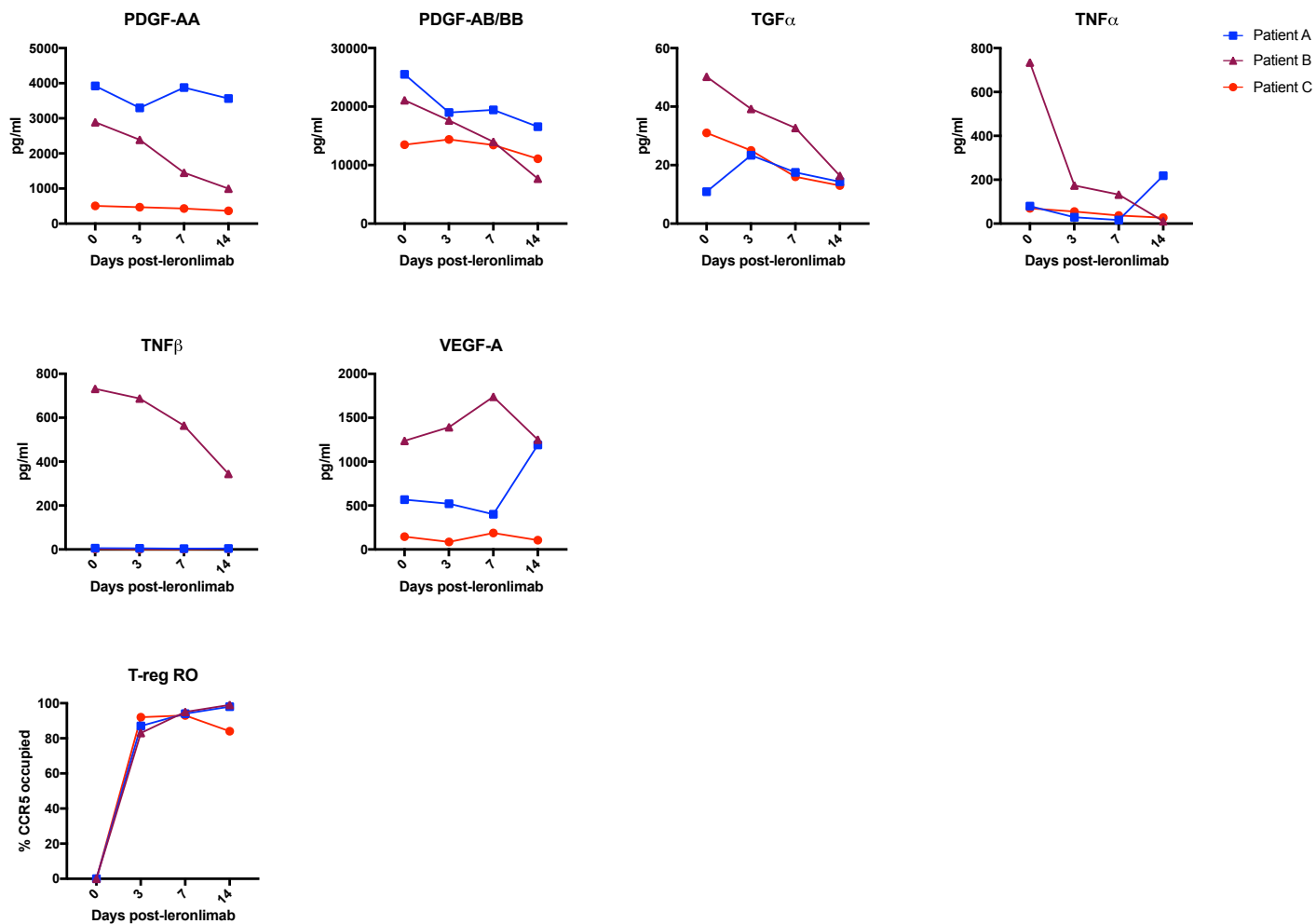

Supplement: Multimedia component 1 [file mmc1.pdf]

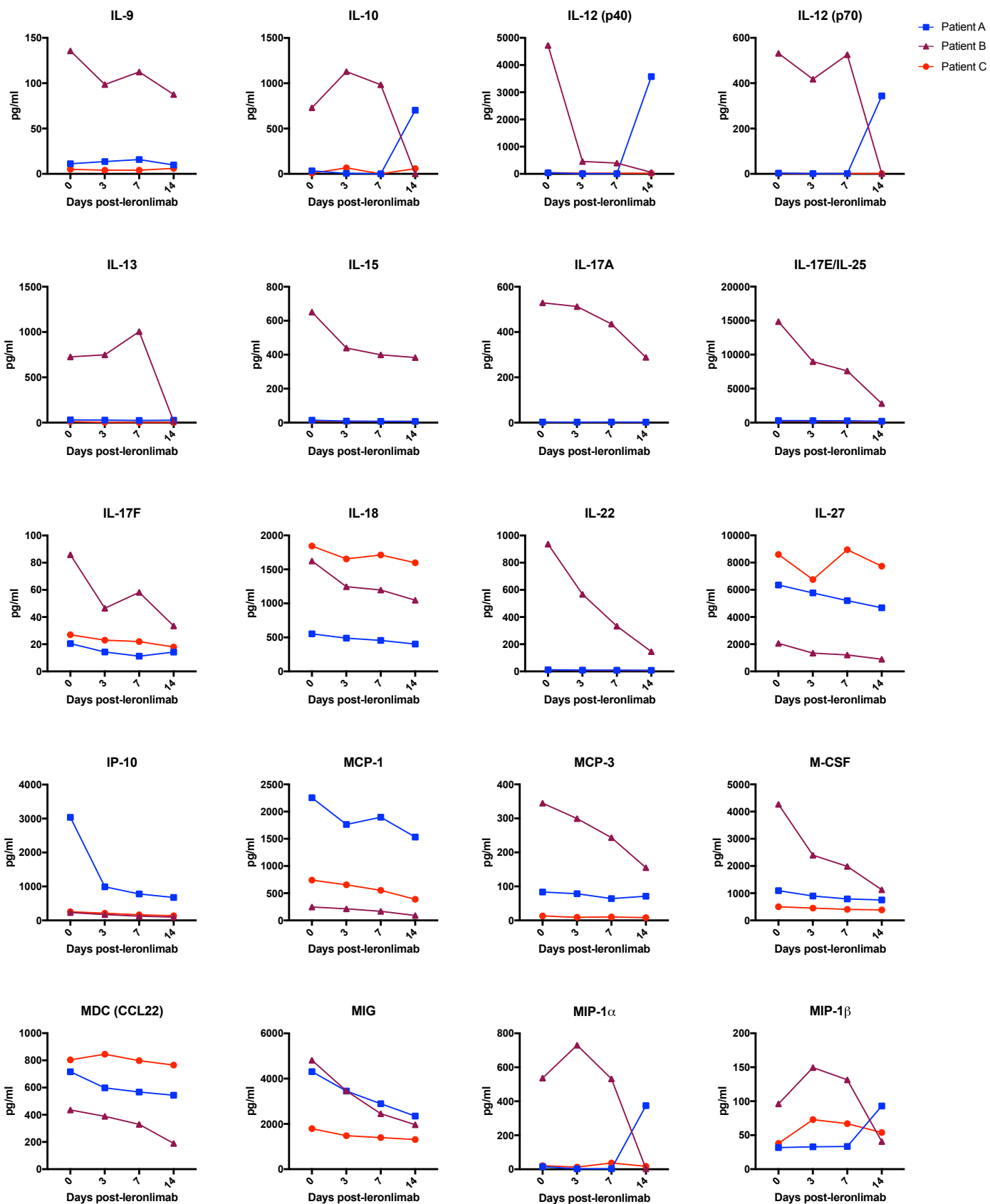

Supplement: Multimedia component 2 [file mmc2.pdf]
